# Vertical Transmission of Extended-Spectrum β-Lactamase (ESBL)-Producing Escherichia coli Infection in Premature Neonates: An Emerging Concern in Developed Countries

**DOI:** 10.7759/cureus.111304

**Published:** 2026-06-22

**Authors:** Pooja S Siddhi, Gireesh Rayasandra, Rohini Thapliyal, Aiden J Plant

**Affiliations:** 1 Paediatrics and Child Health, Walsall Healthcare NHS Trust, Walsall, GBR; 2 Paediatrics, Walsall Healthcare NHS Trust, Walsall, GBR; 3 Microbiology, Black Country Pathology Services, Walsall, GBR

**Keywords:** antimicrobial resistance, early-onset neonatal infection, esbl-producing e. coli, maternal-fetal transmission, necrotising enterocolitis

## Abstract

Early-onset neonatal sepsis (EONS) is predisposed by maternal colonisation and neonatal risk factors. We present two cases of preterm neonates with vertically transmitted extended-spectrum β-lactamase (ESBL)-producing *Escherichia coli* sepsis. In the United Kingdom (UK), the common organisms causing EONS episodes are susceptible to the routinely used first-line empirical antibiotics, i.e., benzylpenicillin and gentamicin. These cases observed in a local neonatal unit highlight the growing concern of drug-resistant *E. coli *infections in this highly vulnerable age group. Gram-negative bacterial sepsis in preterm neonates is associated with long-term morbidity and mortality. Reflecting on our cases, early identification through routine maternal screening in high-risk mothers may enhance clinical suspicion and timely intervention, resulting in better clinical outcomes.

## Introduction

In the United Kingdom (UK), the common organisms causing early-onset neonatal sepsis (EONS) episodes in neonates are group B* *streptococci (GBS) and *Escherichia coli​​​ *[1]. A combination of maternal risks, such as genitourinary colonisation, prolonged rupture of membranes, and maternal bacteraemia, alongside neonatal risks, such as prematurity, predisposes them to EONS. A surveillance study in the UK between 2011 and 2019 reported an increase in the incidence of invasive Gram-negative bacterial EONS (defined as an infection within the first 7 days of life) and late-onset neonatal sepsis (LONS) (defined as an infection >7 days post-birth) [2]. Interestingly, the surveillance reported an unchanged antimicrobial resistance pattern among the common organisms isolated in this age group in the UK during this period [2]. Infections caused by extended-spectrum β-lactamase (ESBL)-producing organisms are harder to treat as they are less susceptible to the routine empirical antibiotics used in hospitals, which include benzylpenicillin and gentamicin, making them life-threatening. The two cases of EONS in preterm neonates observed in a local neonatal unit in the UK are a stark reality check to the dreaded global threat. 

## Case presentation

Case 1

A neonate was born at 32+6 weeks of gestation to an African-origin mother by emergency cesarean section (C-section) for pathological fetal monitoring. A history of premature, prolonged rupture of membranes (PPROM) of 41 hours was recorded. During the pregnancy, the mother was treated for ESBL-producing* E. coli* urine infection, and a high vaginal swab confirmed GBS. Considering the risk factors, the neonate was commenced on first-line intravenous antibiotics, i.e., benzylpenicillin and gentamicin. The blood culture at 7.2 hours grew ESBL-producing* E. coli, *resistant to gentamicin and third-generation cephalosporins; antibiotics were promptly changed to intravenous meropenem (sensitive). At the time of screening, the C-reactive protein (CRP) was 19 mg/L, and the highest recorded value during the course of treatment was 27 mg/L. White cell count was 8.7 x 10^9^/L (normal) and platelet count was 245 x 10^9^/L (Table 1). The cerebrospinal fluid was blood-stained and then cultured, but was negative. Urine culture was negative as well.

**Table 1:** Case 1 - Trend in C-reactive protein during the sepsis episode. WCC: white cell count

Investigations	Day 1	Day 3	Day 5	Reference range
C-reactive protein (mg/L)	19	27	5	<10
WCC (10^9^/L)	8.7	Not done	14.5	5-15
Platelets (10^9^/L)	245	Not done	417	150-400

**Figure 1:**
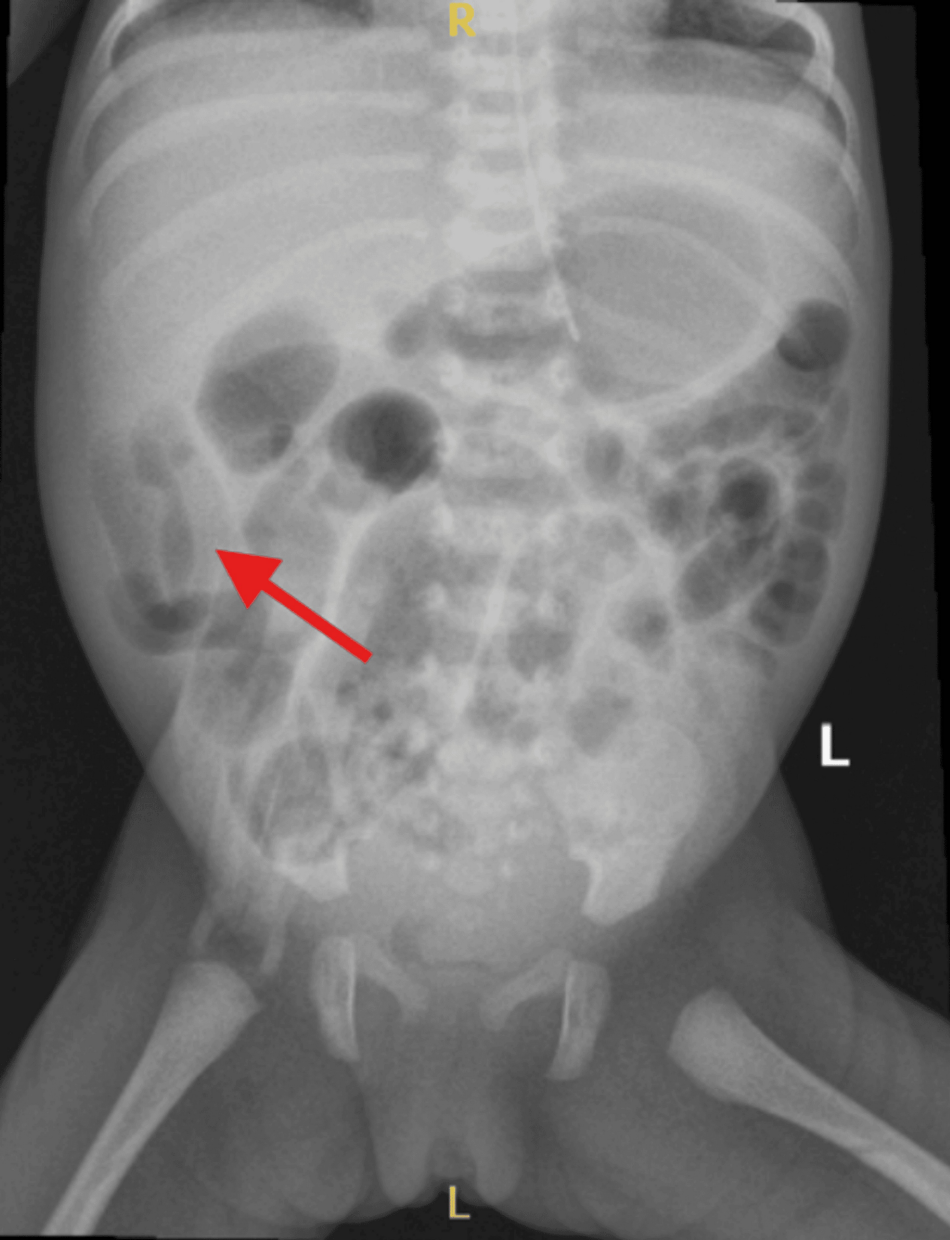
Case 1 - Abdominal X-ray showing dilated bowel loops and no signs of pneumatosis or free air underneath the diaphragm. The red arrow indicates dilated bowel loops.

At 48 hours of life, there were concerns of abdominal distension. Feeds were withheld for 12 hours, and an abdominal X-ray was undertaken. The abdominal X-ray (Figure 1) showed dilated bowel loops and no signs of free air or pneumatosis intestinalis (i.e., evidence of air trapped in the intestinal walls).

The neonate was slowly reintroduced to feeds. The neonate remained well and completed a 21-day course with no infection-related complications. It was discharged following four weeks of in-hospital stay with outpatient follow-up.

Case 2

A neonate was born at 35 weeks to a South-East Asian mother following PPROM of 168 hours. Early pregnancy scans reported abnormal bowel loops in the fetus, which resolved in the later scans. Mother’s antenatal serology and urine cultures were clear throughout the pregnancy. The mother was commenced on prophylactic benzylpenicillin in view of PPROM and preterm labour as per the national guidance [3]. The neonate was clinically well after birth and received enhanced observation for 36 hours per local protocol. The neonate was reviewed the next day by the midwife in the community. The midwife was concerned about the reduced feeding pattern and jaundice. The neonate was referred to the hospital for an urgent clinical review. The neonate was screened for sepsis, and phototherapy was commenced. The admission CRP was 70 mg/L, and the white cell count was 3.4 x 10^9^/L (low) with a normal platelet count (Table 2). Serum bilirubin was 240 micromol/L, above the threshold for phototherapy as per gestational age at birth (in accordance with the National Institute for Health and Care Excellence (NICE) guidance) [4].

**Table 2:** Case 2 - Blood investigations on admission. WCC: white cell count

Investigations	On admission	Reference range
Hemoglobin (g/dL)	194	140-200
WCC (10^9^/L)	3.4	5-15
Platelets (10^9^/L)	203	150-400
Neutrophils (10^9^/L)	2.0	2-8
Lymphocytes (10^9^/L)	1.2	2-11
C-reactive protein (mg/L)	70	<10
Serum bilirubin (micromol/L)	240	-

**Figure 2:**
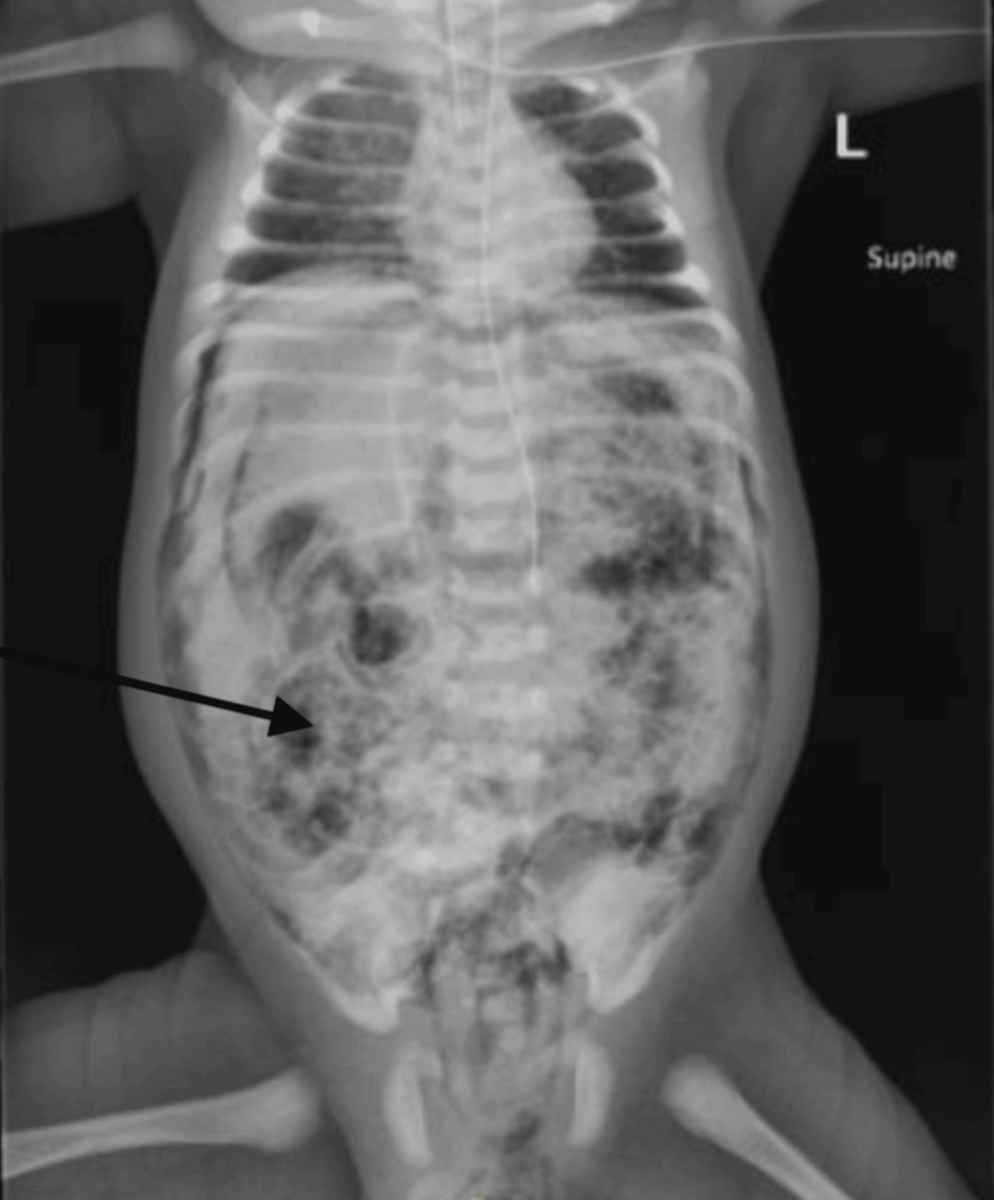
Case 2 - Abdominal X-ray demonstrating extensive pneumatosis intestinalis, i.e., cystic lucency in the walls of the intestines and evidence of free air in the abdomen. The black arrow indicates pneumatosis intestinalis and free air underneath the diaphragm.

The neonate developed significant abdominal distension within the first six hours of hospital admission. The team suspected necrotising enterocolitis (NEC) and commenced triple antibiotics, i.e., cefotaxime, teicoplanin, and metronidazole. The abdominal X-ray revealed extensive pneumoperitoneum and features suggestive of NEC, such as portal gas (Figure 2). This sudden deterioration was reflected in the blood parameters, which showed a further drop in the white cell count to 2.9 x 10^9^/L and platelets to 110 x 10^9^/L. The neonate was transferred urgently to a surgical centre. ESBL-producing *E. coli *was cultured from blood within 14.4 hours of incubation. The ESBL-producing *E. coli *isolated was sensitive to meropenem and resistant to gentamicin and third-generation cephalosporins. This information was communicated to the retrieval team, and the antibiotics were changed to meropenem. An emergency laparotomy revealed widespread inflammation and necrosis of the intestines. The care was reoriented to palliation with parental agreement. The neonate died on day 4 of life in comfort care.

## Discussion

ESBL-producing organisms have the inherent ability to hydrolyse the β-lactam ring of broad-spectrum β-lactams, including third-generation cephalosporins. One of the mechanisms of ESBL resistance transmission in Gram-negative bacteria is plasmid-mediated, i.e., resistance pattern transferred horizontally between bacterial species. The plasmid-mediated resistance has additional ammunition that can confer resistance to cephalomycins and β-lactamase inhibitors. In addition, ESBL-producing bacteria frequently exhibit broad co-resistance to aminoglycosides and quinolones because the genes encoding these resistances are often housed together on the same mobile genetic elements, such as plasmids and transposons. Consequently, plasmid-mediated resistance is on the rise.

ESBL-producing organisms are common as hospital-acquired infections; nonetheless, an increasing carriage among healthy individuals in the community has been identified worldwide. A rising trend in ESBL-producing organism colonisation has also been reported in neonatal units globally. Molecular surveillance screening in neonatal units for ESBL-producing organisms at two centres in Kenya and Nigeria reported up to 85% colonisation in neonates [5]. A single-centre observational prospective study from Mexico reported 87% colonisation in neonates, of whom 29% developed signs of infection [6]. The duration of hospitalisation, presence of invasive devices, and prolonged antibiotic courses contributed to episodes of infection/sepsis [6].

These two neonatal presentations of Gram-negative bacteraemia in our hospital were early-onset in nature (infection within 7 days of life) and shared a few other commonalities between them, including preterm birth (<37 weeks), PPROM, and mothers originating from countries with reported high prevalence of ESBL-producing *E. coli* in the community. Although in the second case no maternal microbiological samples were suggestive of prior ESBL-producing* E. coli *colonisation, the early-onset nature and the absence of other risks, such as prolonged in-hospital stay, previous or prolonged antimicrobial use, and the presence of invasive devices and procedures, suggest vertical transmission as the likely cause.

Vertical transmission of ESBL-producing *E. coli* has been under surveillance in some countries. A prospective cohort study conducted in 2015-16 in Israel identified carriage rates of 21.5% in mothers and 14.8% in neonates; of them, 5.6% (n=4) developed late-onset sepsis, and two died [7]. The colonisation in mothers and neonates was confirmed with rectal swabs. Twenty-five mother-neonate pairs were colonised with the same bacterial strain. The significant risk factor for ESBL-producing *E. coli* colonisation was observed to be prematurity (odds ratio (OR): 1.33, 95% confidence interval (CI) 1.01-1.75, p=0.041) [7]. A prospective study in Indonesia is underway (NeoCol) that aims to evaluate the timing and acquisition of multidrug-resistant bacterial colonisation in mothers and babies in the community. This study also aims to study the antimicrobial use and its impact on the neonatal intestinal microbiome [8]. 

A recent multicentre NeoSEAP (Neonatal Sepsis in South East Asia) study reported a higher incidence of Gram-negative bacterial infections in neonates, revealing alarming levels of non-susceptibility to the commonly prescribed empirical antibiotics [9]. Among the Gram-negative bacteria, the pooled susceptibility to the recommended empirical antibiotics, such as benzylpenicillin/ampicillin plus gentamicin or third-generation cephalosporins, was less than 50% [9]. A UK surveillance study (2011-2019) reported an increasing trend in* E. coli-*associated early-onset sepsis episodes. *E. coli* resistance to gentamicin/amikacin/tobramycin increased from 5.8% to 6.8% (p<0.001) during this surveillance period [2]. Another study on characterisation of antimicrobial resistance in Gram-negative bacterial isolates in neonatal sepsis from low- and middle-income countries reported 67% (597/885) resistance to at least one β-lactam and one aminoglycoside among the Gram-negative isolates [10]. With the growing antimicrobial resistance worldwide and the increasing use of carbapenems in managing neonatal sepsis episodes, we have set ourselves up for an even greater challenge and may eventually run out of antibiotics. Hence, the question of what comes next.

The NeoSep 1 trial has commenced a prospective study on the safety and efficacy of the combination drugs - fosfomycin-amikacin, flomoxef-amikacin, and flomoxef-fosfomycin - in hospitals in Kenya and South Africa [11]. This study aims to prospectively recruit 3000 neonates (<28 days of age) from the study sites and has two study parts. The first part is a pharmacokinetic study of the newer antimicrobials, and the second part assesses the efficacy of the test antimicrobials in treating the infections and preventing deaths [11]. This study is funded and aided by the World Health Organisation (WHO) and the Global Antimicrobial Research and Development Partnership (GARDP), prioritising this highly vulnerable group born in high-resistance incidence countries. As we step into a whole new decade of the fight against antimicrobial resistance, the outcomes from such studies will play a pivotal role in guiding national policymakers and clinicians in making the right antibiotic choices for treating infections in early life.

## Conclusions

In the current UK setting, the incidence of ESBL-producing* E. coli *neonatal sepsis episodes is very low. However, a constant vigilance of the worldwide trends of antimicrobial resistance is indispensable. Strategies to improve maternal and neonatal surveillance that support early identification of drug-resistant organisms and timely intervention should be considered. With many promising maternal and neonatal studies in high drug-resistance regions underway - exploring the timing of colonisation, risks leading up to neonatal infections, and pharmacokinetics of newer antimicrobial combinations - undeniably, we are heading toward a never-ending cascade of drug resistance. 
